# Evolution of anatomic pathology workload from 2011 to 2019 assessed in a regional hospital laboratory via 574,093 pathology reports

**DOI:** 10.1371/journal.pone.0253876

**Published:** 2021-06-29

**Authors:** Michael Bonert, Uzma Zafar, Raymond Maung, Ihab El-Shinnawy, Ipshita Kak, Jean-Claude Cutz, Asghar Naqvi, Rosalyn A. Juergens, Christian Finley, Samih Salama, Pierre Major, Anil Kapoor

**Affiliations:** 1 Pathology and Molecular Medicine, McMaster University, Hamilton, Ontario, Canada; 2 Internal Medicine, McMaster University, Hamilton, Ontario, Canada; 3 Pathology, University of British Columbia, Vancouver, British Columbia, Canada; 4 Medical Oncology, McMaster University, Hamilton, Ontario, Canada; 5 Thoracic Surgery, McMaster University, Hamilton, Ontario, Canada; 6 Urology, McMaster University, Hamilton, Ontario, Canada; University of Waterloo, CANADA

## Abstract

**Objective:**

Quantify changes in workload in relation to the anatomic pathologist workforce.

**Methods:**

In house pathology reports for cytology and surgical specimens from a regional hospital laboratory over a nine- year period (2011–2019) were analyzed, using custom computer code. Report length for the diagnosis+microscopic+synoptic report, number of blocks, billing classification (L86x codes), billings, national workload model (L4E 2018), regional workload model (W2Q), case count, and pathologist workforce in full-time equivalents (FTEs) were quantified. Randomly selected cases (n = 1,100) were audited to assess accuracy.

**Results:**

The study period had 574,093 pathology reports that could be analyzed. The coding accuracy was estimated at 95%. From 2011 to 2019: cases/year decreased 6% (66,056 to 61,962), blocks/year increased 20% (236,197 to 283,751), L4E workload units increased 23% (165,276 to 203,894), W2Q workload units increased 21% (149,841 to 181,321), report lines increased 19% (606,862 to 723,175), workforce increased 1% (30.42 to 30.77 FTEs), billings increased 13% ($6,766,927 to $7,677,109). W2Q in relation to L4E underweights work in practices with large specimens by up to a factor of 2x.

**Conclusions:**

Work by L4E for large specimens is underrated by W2Q. Reporting requirements and pathology work-up have increased workload per pathology case. Work overall has increased significantly without a commensurate workforce increase. The significant practice changes in the pathology work environment should prompt local investment in the anatomic pathology workforce.

## Introduction

Workload assessment in pathology has evolved with changes in pathology practice and is typically divided into “technical” and “professional”; the latter is done by the pathologists and is the focus of this work.

Traditional measures of the professional component of pathology workload include: number of cases (surgical specimens, cytology specimens, autopsies), number of blocks and number of slides; however, these have been found to be poor representations of actual work [1]. Thus, many workload systems have been proposed and are currently in use, e.g. Work2Quality (W2Q) [2], CAP-ACP Workload Model (Level 4 Equivalent) [3], Royal College of Pathologists United Kingdom [4]. In a fee for service context/capitation model of compensation, fee codes (e.g. Current Procedural Terminology (CPT) of the United States, Ontario Schedule of Benefits in Canada) can be considered a type of workload model.

Prior work suggests that appropriately weighted specimens best reflect professional workload [1]. This is important as workload and work distribution have a significant impact on patient safety, as a high workload in pathology is associated with poor quality and medical errors [5–7]. If a mechanism to adjust for workload changes is lacking, in the context of salaried pathologists and increasing work, the result may be excessive work/chronic under-staffing and poor service.

The practice of pathology has shifted dramatically in the past ten years. Reporting requirements have increased, and many elements are mandated by regional, national and international organizations and also by payers and/or tracked as quality metrics. In the Province of Ontario, electronic synoptic reporting was implemented over 10 years ago [8]. Ontario laboratories get monthly reports from the government showing rate of synoptic reporting. In the State of California, electronic synoptic reporting has been mandated by law since 2019 [9]. In addition, recent advances in molecular pathology and advanced diagnostics with mandated tests to guide cancer management have increased pathologist work.

Optimal resource allocation is predicated on (1) needs, (2) vantage point (payer versus provider) and (3) value (efficiency). On value, providers’ and payers’ interests often align. On needs, providers and payers may be diametrically opposed; providers want more—payers may say “funding is sufficient”. Objective workload data that is representative of the work done in this context is useful and (if properly collected and interpreted) may be an objective way to (1) mediate competing demands, and (2) service the needs patients and other stakeholders optimally.

This work analyzes the reports of the regional laboratory over a nine-year period (2011–2019) to assess changes in (1) reporting and (2) gauge workload in relation to the workforce. The analysis makes use of two competing workload systems (the nationally endorsed CAP-ACP Workload Model (2018 Update) versus the regionally endorsed Work2Quality), and a set of billing codes (Ontario Schedule of Benefits—March 2020).

The Work2Quality system and Ontario Schedule of Benefits (SOB) is based on 6-tiers in surgical pathology (somewhat similar to the Current Procedural Terminology (CPT) codes 88300, 88302, 88304, 88305, 88307, 88309)–see Appendix “Overview of Workload Systems”. The SOB codes (L861, L862, L863, L864, L865, L866) are readily accessible within the pathology reports; thus, they were used to stratify the data. The existence of a third system formal workload system (AABACUS) in our jurisdiction was noted [10, 11]. It was not implemented in this study as the published technical description of the workload system was limited.

The work also examines parameters often discussed in the context of workload (case count, blocks, ancillary tests (e.g. immunohistochemical stains, special stains), formal and informal consultations).

## Materials and methods

Ethics approval was obtained (Hamilton Integrated Research Ethics Board (HiREB) identifier: #4879) to examine all pathology reports for cases accessioned January 1, 2011 to December 31, 2019 from the regional laboratory that encompasses Hamilton General Hospital, St. Joseph’s Healthcare Hamilton, McMaster University Medical Centre and Juravinski Hospital, and assess the workload. Consent to analyze the data was not applicable as the data was fully anonymized.

All pathology reports were extracted from the laboratory information system (MEDITECH) in the in house “final” format. The “final” format contained coded laboratory procedures for most ancillary tests and the block count. Following report extraction, the laboratory information system played no role in the further data processing or the analysis. Following the data extraction, custom computer code, written in Python (https://www.python.org) removed all the patient identifiers. Further data processing was done with a suite of programs written in Python developed from a previously written code base [12].

The suite of computer programs reconstructed the report structure in the laboratory information system. Using knowledge about the report format and the coded procedures within the report, cases were classified with a hierarchical string-matching algorithm and report elements were extracted. The free text elements and pathology procedures were parsed to generate the case workload units as per Level 4 Equivalent 2018 (L4E) and Work2Quality (W2Q) systems as well as calculate the total (Ontario) Schedule of Benefits fees (SOBF). The block count, number of lines and characters in the diagnosis section, microscopic section, and cancer care summary (synoptic report) and sign out date were extracted.

These programs also irreversibly anonymized the submitting physicians and standardized pathologist names, generated a preliminary anonymized tabulation of the cases, generated an anonymized random subset of data that could be used for auditing in LibreOffice Calc [13], and coded all cases into a format that could be read by R [14].

The L4E coding was audited using the randomly selected anonymized cases, by five pathologists, and the results used to refine the suite of programs. A script in R further processed the data and was used to do statistical analysis, normalizations, tabulations, and plots. The R script extracted the sign-out date and converted it to the International Standards Organization (ISO) 8601 (standardized) week, as implemented in the R ‘strptime’ package. The ISO 8601 week was chosen as (1) it is a well-established standard, and (2) defines a week as starting on Monday and ending on Sunday.

Full-time equivalent (FTE) pathologists (clinical service only) were defined in this analysis as those who signed cases in >41 ISO 8610 weeks per calendar year; they were considered 1.0 FTE. If the pathologist signed cases in <42 ISO 8610 weeks per calendar-year the FTE was calculated as: FTE = (ISO 8610) weeks signing/48 weeks signing; example: a pathologist signed cases in 24 weeks of the year—they would be: 0.5 FTE (24/48 = 0.5). The forty-two weeks cut point was chosen to define full-time as full-time pathologists can have up to eight weeks vacation and two weeks continuing medical education per year. The forty-eight week cut-point was chosen as temporary pathologists typically receive three weeks vacation and one week continuing medical education per year.

Immunohistochemical stains were captured by the number of HR (heat retrieval) codes. This methodology captured negative immunostaining controls done early in the study period.

### CAP-ACP workload—2018 update (Level 4 Equivalent 2018)

The Level 4 Equivalent system is described in a document on the CAP-ACP website [3]. The implementation of L4E scoring included fragment counting and core counting for biopsies and block counting for large excisions to indirectly capture amount of work. This was done by parsing the ‘source of specimen’ section of the report (which captures how the submitting MD labelled the container bottles) and the ‘gross pathology’ section of the report.

The adjustment for “micro-only” was done, as the grossing in the environment is done by pathology assistants and pathology residents. This adjustment was done (on the “base L4E”) before adding the (L4E) points for the intraoperative consultations (frozen sections), ancillary testing (special stains, immunohistochemical stains, molecular testing), synoptic reports and consultations. The discount was based on the L4E 2018 definition; however, it was simplified. Cases with a “base L4E” greater than 10 L4E units (or 1 L6 unit) were reduced in value (or “discounted”) by 10%. Cases with less than 10 L4E units were discounted 5%.

Informal consultations (involving a few slides or review of a small case) were captured by searching the diagnosis and microscopic sections of the report for “Dr.” and “DR.” to capture unique individuals. If one doctor was found: 0.5 L4Es were added to the case. If two or more doctors were found: 1.0 L4Es were added to the case. If the case had a formal consult, i.e., the report had a “consultation” section, the value of the case was multiplied by 1.5x. As the institutions forming the regional laboratory are teaching hospitals with pathology residents, the L4E was adjusted; as per the L4E (2018) manual the adjustment is 1.3x.

### Work2Quality (W2Q) and schedule of benefits fees

The implementation of W2Q was primarily based on the “pathology procedures” section of the report and the “source of specimen” section of the report. The W2Q score included adjustments for internal and external consults, special stains, immunohistochemical stains, molecular testing, synoptic reports, and frozen sections. An adjustment for teaching was not included.

The March 2020 Ontario Schedule of Benefits fees [15] were calculated using the parameters from the W2Q analysis. Currency amounts are all in Canadian dollars. Formal and informal consults were not captured for in the Schedule of Benefits fees (SOBF) calculation.

### Classification by L86x codes

Cases may have multiple codes, e.g., a case may simultaneously have a L864 code and a L866 code. Codes are applied to the parts of a specimen, i.e., each container gets a code. The W2Q calculation and the SOBF calculation counted all L86x codes.

Cytology cases with a cell block are coded within the laboratory as L864 plus the relevant cytology code (e.g. L805). For the purpose of classifying cases, the highest L86x code was used if several were present. For example, a case with L866 and L864 would be classified as L866, and a case with L864 and L863 would be classified as L864. Cytology cases without a cell block were classified as ‘L86x = = 0’; this group was defined by no L86x code being assigned to the case.

## Results

The practice has a multitude of case types (surgical, non-gynecologic cytology, gynecologic cytology, cancer reviews, external consults, autopsies) and include a range of ancillary tests and intra-operative consultations (so-called “frozen sections”).

A preliminary analysis of the reports demonstrated that the cancer reviews (CR) and external consults (RR) do not contain all the parameters in the other reports; thus, these cases were excluded from the analysis. The CR cases were approximately 700–800 cases/year on average and a relatively small subset of the reports over all. The RR cases are a larger subset; on average there were approximately 3500 RR cases/year. Fetal autopsies (accessioned with surgical case identifier) were included in the analysis. Other autopsies (labelled with a unique autopsy identifier) were excluded from the analysis.

All other cases (in house surgical cases from four hospitals, in house (non-gynecologic) cytology cases from three hospitals, and in-house gynecologic cytology cases from two hospitals) were deemed suitable for analysis; in total, these were 574,099 pathology reports. Data could be extracted from 574,093 pathology reports. The study period included reports signed out by a total of 63 different pathologists. Randomly selected reports were assessed by five pathologists for the L4E (2018) scoring. The L4E (2018) coding accuracy in 1,100 cases was 95%.

The L4E units, W2Q units, (Ontario) SOBF, block count, number of report lines (diagnosis, microscopic and cancer care summary), immunohistochemical stains (IHCs), special stains, FTEs and cases were captured as shown in Table 1.

**Table 1:** Total laboratory workload and workforce in full-time equivalents.

Year	L4E (2018)	W2Q	SOB Fees	Blocks	Report Lines	IHCs	Spec. Stains	FTEs	Cases
2011	165276.0	149840.5	$6,766,927.15	236197	606862	40336	15199	30.42	66056
2012	181625.2	159131.7	$7,122,570.15	259095	649932	48448	15860	29.99	67447
2013	186130.0	162329.4	$7,206,866.00	269501	622968	62909	14857	30.18	64039
2014	190637.1	171949.7	$7,612,328.20	274096	622560	79984	14660	30.02	63417
2015	199294.6	178304.4	$7,840,372.25	285925	648349	84132	14559	29.81	65003
2016	196077.6	174116.9	$7,613,806.30	281975	670842	74353	14319	30.32	64671
2017	193285.0	174307.7	$7,604,699.65	281910	677908	70251	14102	30.73	61134
2018	199897.5	176058.5	$7,551,597.25	287437	694060	65023	13165	30.96	60364
2019	203893.6	181321.5	$7,677,109.20	283751	723175	60423	14140	30.77	61962

L4E = Level 4 Equivalent workload system (2018 definition); W2Q = Work2Quality workload system; SOB fees = (Ontario) Schedule of Benefits (total) fees; Blocks = total number of blocks; Report Lines = number of lines in the ‘diagnosis’ section, ‘microscopic’ section and synoptic report (Cancer Care Summary); IHCs = number of immunohistochemical stains (as per ‘heat retrieval’ code), Spec. Stains = number of special stains; FTE = full-time equivalent anatomical pathologists; Cases = total number of in house cases (surgical + cytology).

**Fig 1:**
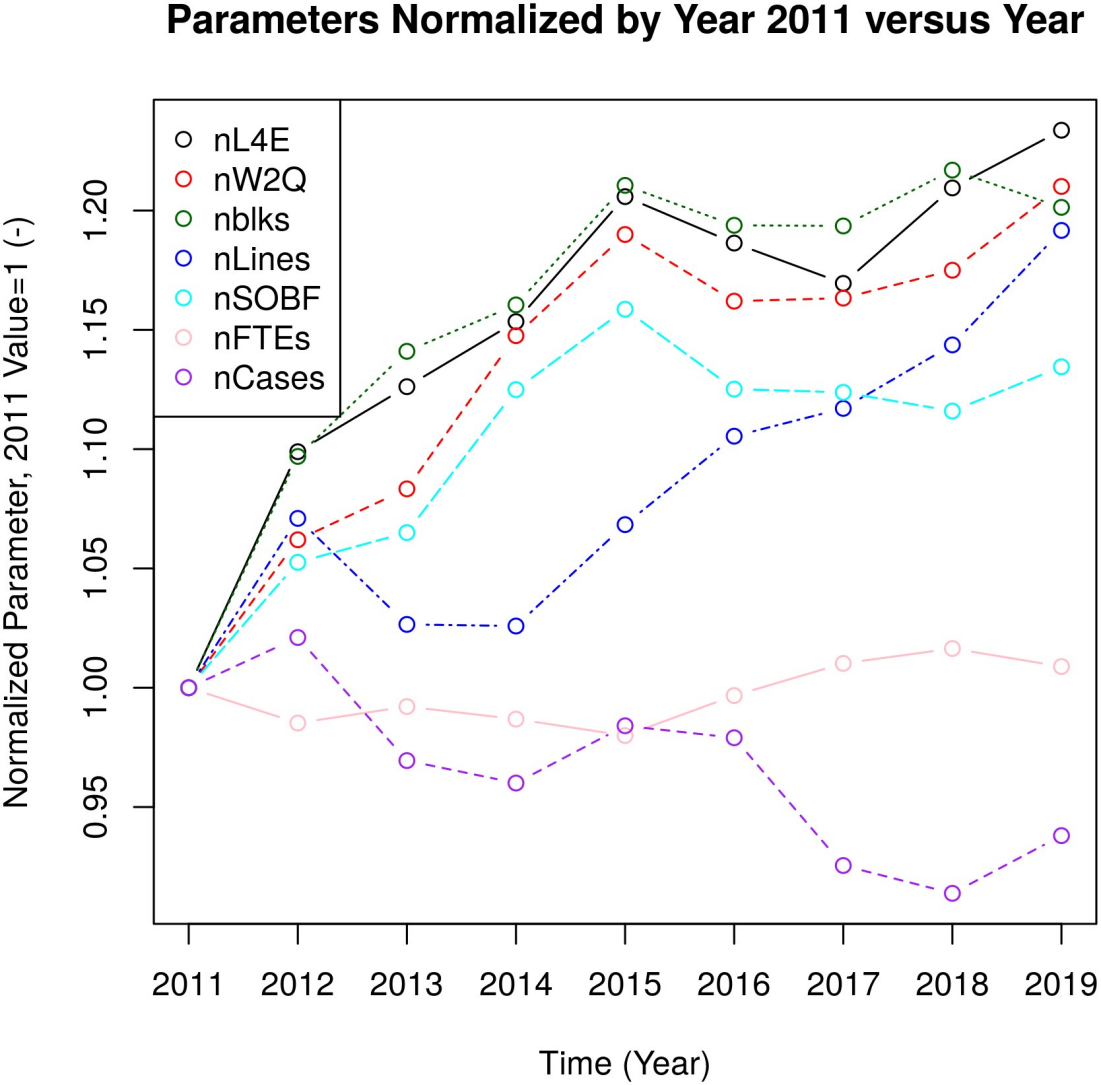
Parameters over time with 2011 as the reference point. nL4E = normalized Level 4 Equivalent units (2018 definition); nblks = normalized number of blocks; nW2Q = normalized Work2Quality units; nSOB fees = normalized (Ontario) Schedule of Benefits fees, nLines = normalized number of lines in the ‘diagnosis’ section, ‘microscopic’ section and synoptic report (Cancer Care Summary); nFTE = normalized full-time equivalent anatomical pathologists; nCases = normalized number of in house cases (surgical + cytology).

Immunostain use increased in 2011 from 40,336 to 84,132 in 2015; however, it decreased steadily thereafter and, in 2019, was 60,423. Special stain use was relatively flat through the study period; 15,199 stains were done in 2011. In contrast, in 2019, 14,140 special stains were performed. The data was normed to the 2011 values, and selected values were plotted as shown in Fig 1.

A sub-analysis on the block count per case stratified by L86x category showed minimal to moderate increases; the blocks/cases changed +13% for L861/2/3, +2% for L864, +14% for L865 and +13% for L866 over the study period (2011–2019). A sub-analysis that examined the number of lines in the report demonstrated that the ‘diagnosis’ section and ‘CCS’ section increased in length, and the ‘microscopic’ section decreased modestly in length. Overall, the length of pathology reports increased (Table 2).

**Table 2:** Report lines.

Year	Dx	Micro	CCS	Sum
2011	407597	104095	95170	606862
2012	422247	80250	147435	649932
2013	400027	77735	145206	622968
2014	408174	64153	150233	622560
2015	429078	62504	156767	648349
2016	448605	66375	155862	670842
2017	451190	62156	164562	677908
2018	445290	57564	191206	694060
2019	447975	80901	194299	723175

Dx = diagnosis section of report; Micro = microscopic section of report; CCS—Cancer Care Summary (synoptic report), Sum—the sum of Dx, Micro and CCS.

The synoptic report section, formally known as ‘Cancer Care Summary’ (CCS), has more than doubled over the study period. In 2019, it exceeded 40% of the ‘diagnosis’ section. An analysis that considered the number of characters in the report was also done. It demonstrated the same trends as the analysis based on the number of lines.

The fees per workload was significantly higher for biopsies and decreased over the study period. Dollars per workload unit (L4E) decreased from $40.94 to $37.65. The compensation per workload unit has decreased in all L86x categories. Larger decreases were seen for biopsies (L864 fee/L4E unit: $55.53 to $48.01) than large resections (L866 fee/L4E unit: $29.62 to $27.14). The yearly billings for L861-L863 were lumped as they were modest in relation to other categories; the average total yearly billings over the study period were $88,158/year for L861-L863, $4,805,908/year for L864, $1,394,621/year for L865, $871,190/year for L866 and $284,153/year for L86x = = 0. Cases were stratified by the L86x codes and summary statistics were calculated. The median L4E, median W2Q and median SOB fees by L86x are shown in Table 3.

**Table 3:** Median values by L86x classification.

L86x Classification	L4E (2018)	W2Q	SOB Fees	Specimen Type	Examples
L861/L862/L863	0.6175	0.29	$14.30	small resections	appendectomy, ganglion cyst
L864	1.8525	2.00	$97.30	most biopsies	breast core biopsy, stomach biopsies
L865	5.135	4.04	$165.60	intermediate size resections	benign hysterectomy, salivary gland resection
L866	15.32375	8.29	$347.25	large resections	radical prostatectomy, laryngectomy
L86x = = 0	1.235	0.56	$4.60	uncomplicated cytology	Pap test, urine cytology

L861/L862/L863 lumps cases that have at one of those codes and no L864 or L865 or L866 codes; L864 includes all cases with at least one L864 code and no L865 or L866 codes; L865 includes all cases with at least on L865 and no L866 code; L866 includes all cases with at least one L866 code; L86x = = 0 includes cases without any L86x codes—these are cytology cases without cell blocks.

## Discussion

The custom computer code could reliably extract elements from the reports. Case classification and workload measure determinations were likely sufficiently accurate to infer significant trends. The anonymized data used in this analysis is available for review (see supplemental materials).

Subjectively, pathologists have felt work increase. Yet, in an apparent paradox, the traditional unit of workload measurement (number of cases) suggests work has actually decreased. The data herein indicates quite clearly that workload has increased.

### Block count and number of lines

The analysis herein shows that the report length has increased and the number of blocks increased. The length per report was not explicitly assessed; however, as the number of reports decreased, it should be apparent that the report length (as assessed by ‘diagnosis’, ‘micro’ and ‘CCS’) increased >20% per case. The number of blocks increased because of (1) a shift away from cytology specimens, and (2) an increase of blocks per case, in selected types of cases.

The length of ‘diagnosis’, ‘microscopic’ and ‘CCS’ sections were evaluated, as they are the bulk of what the pathologist writes/dictates/enters, if they are not grossing. The report length is most certainly increasing universally (i.e. outside of the laboratory studied), as it is likely driven by (mandated) reporting requirements. The block counts also appear to be increasing universally, i.e. outside of the regional laboratory [16].

### Synoptic reports

Pathologists have benefited from synoptic reporting; however, it has not been without considerable effort—that arguably may exceed the benefits to them. Seen in a larger context, it should be noted that: oncologists, surgeons and epidemiologists have benefited from the synoptic reporting; completeness and satisfaction with reports has increased [17]. In this context, when compared to free text, the synoptic report is, in the economics lexicon, an *externality*; pathologists deliver more value (reports are more complete, standardized, contain more information, easier to analyze) without more cost to the individuals consuming them. Thus, it can be argued that a re-balancing/further investment in pathology is needed, as pathologists are delivering more information and more value. Seen from a system management/research perspective, capturing data in individual fields is preferable in medicine, as it facilitates analysis work by reducing cost. If healthcare providers are required to absorb this cost, adoption will likely be slower.

### Limitations

The reports do not capture all workload elements consistently. For example, the number of blocks is not always recorded in the pathology procedures section of the report; a sub-analysis showed that 1,716 (of 406,719) surgical cases are defective in this regard. Based on the block count sub-analysis and the audited cases (n = 1,100 cases), it is estimated that workload parameters in the reports have an error or are incomplete in 1–2% of cases.

A small number of cases had apparent coding errors that led to extreme workload/SOBF values (e.g. one case had 442 HR codes—but was truncated to two digits); these prompted case maximum values. The number of L4E units was limited to maximum of 50 per case. The number of W2Q units was limited to 50 per case. The SOB fees were limited to $1,500 per case. On re-calculation the maximums resulted in a very small but noticeable correction. The L4E, W2Q and SOBF maximums were seen in 152, 84 and 395 cases.

Employment records from human resources were not available (to the authors) to assess the workforce. Thus, pathologist work was assessed based on the final signature only. If a pathologist only signed addenda or did not sign-out cases during a given ISO 8601 week it would not be counted. The ISO 8601-week definition de facto assumes that the pathologists take their continuing medical educational (CME) leave/vacations in whole weeks (off work Friday/return to work Monday). If a pathologist customarily takes their vacations or CME Wednesday to Tuesday, the analysis done herein under-counts the weeks off/over-counts the weeks worked. With the FTE definition used, this is a potential issue for (1) part-time pathologists, i.e. pathologists that signed less than 42 ISO weeks in a year, and (2) pathologists that are at the full-time/part-time interface (41 weeks versus 42 weeks). For pathologists clear of the 41-week cut-point there is no difference from a week more of less, e.g. it makes no difference whether a pathologist is signing for 44 weeks or 45 weeks.

The molecular tests were not separately tabulated/counted; however, they were captured in the workload assessment. A simple count of all the molecular tests is likely non-trivial to interpret, as there are a number of different tests (e.g. sequencing (multiple genes versus single genes), FISH, SISH).

The analysis does not consider the time value of money or the Ontario Schedule of Benefits changes. A more complex analysis would adjust for the Ontario Schedule of Benefits changes and costs of living increases that took place over the time period analyzed. As physicians services compensation increases have been modest in comparison to the cost of living changes [18–20], a more rigorous analysis taking this into account would likely increase the negative compensation per work unit trend. As presented, the analysis (in 2020 dollars) is likely a conservative calculation that forgoes some accuracy for lower complexity.

### Evolution of practice and fee codes

The fee codes heavily value biopsy specimens. We believe this is largely due to history; the codes were created when “big” specimens (L866 cases) were typically “smaller” (<10 blocks), the number of diagnostic lines less (frequently <5 lines), biomarkers (e.g. HER2 testing) did not exist, and Cancer Care Summaries (synoptic reports) did not exist.

The data herein shows that the ground is shifting; the compensation per unit work is changing dramatically. At the current point in time, the subset of pathologists that do primarily small specimens (L864) are considerably advantaged. Whether small specimens should be more highly valued and if so by how much, should be further considered; however, this is beyond the scope of this work.

### Level 4 equivalent (L4E 2018) system

The L4E system is complex and was a challenge to implement with the current reporting practices. The increase in work, as measured by L4E units, was driven by synoptic reporting, increased block counts, and a shift to larger specimens. Several aspects of the L4E system were not implemented, e.g. the increased workload points for high-grade dysplasia in polyps (as was likewise excluded by Halwani et al. [21]), clinician consult, case conferences with clinicians (as this information is not consistently well captured in reports).

The immunostain use drove some increases in the time frame 2011 to 2015, as the number of immunohistochemical stains increased in the time frame. The decrease in IHC use in the later part of the period was driven by several factors. The large factor was likely the elimination of negative immunostain controls, in the context of polymer-based antigen retrieval (as recommended by the Institute for Quality Management in Healthcare [22]). A subset of cases were previously analyzed in detail in a separate study; this likely led to some decreases, due to changes in practice [23]. Also, staff turnover is suspected to have played a role.

### Work2Quality (W2Q)

The W2Q system, like the L4E system, shows increased work; however, the change is less pronounced. Ancillary tests are more heavily weighted in the system. The significant decline in IHC tests and a dramatic shift away from L86x0 cases and a relative shift away from L864 cases (due to gynecologic cytopathology and small gynecologic specimens moving to another laboratory) counteract (1) the increased reporting requirements and (2) increased molecular testing.

### Comparing W2Q and L4E 2018

W2Q and L4E yield a similar work increase; however, this appears to be a chance finding that is due to several simultaneous changes. To illustrate this, we calculated differences between hypothetical practices that purely do L864 cases and L866 cases, using the medians calculated in the data set. If one compares a pure L864 practice (an environment that only does L864 cases) with a pure L866 practice (an environment that only does L866 cases) on the basis of a yearly target of 7,560 L4E units per pathologist, the SOB fees would be $397,089.50 (7,560 L4E x $97.30 / 1.85 L4E = $397,089.50) and $171,316.40 (7,560 L4E x $347.25 / 15.43 L4E = $171,316.40) respectively; the difference is $224,762.10 for the same work in L4E units.

If one uses a yearly W2Q target of 7,500 W2Q units per pathologist, the SOB fees would be $364,875.00 (7500 W2Q x $97.30 / 2.00 W2Q = $364,875.00) and $314,158.60 (7500 W2Q x $347.25 / 8.29 W2Q = $314,158.60) for pure L864 and L866 practices respectively; the difference (favouring L864 practices) is $50,716.38 for the same W2Q units. If one recalculates the work 7,500 W2Q units into L4E units, the work would be 6946.875 (7500 W2Q x 1.85 L4E / 2.00 W2Q = 6946.875) and 13,863.47 (7500 W2Q x 15.43 L4E / 8.29 W2Q = 13,863.47) for the pure L864 practice and pure L866 practice respectively; the difference is a factor of 2.0x.

The systems (L4E and W2Q) give dramatically different results for different environments. W2Q favours small specimens (that are done in non-hospital labs in Ontario) and disadvantages environments with large specimens (hospital pathologists and academic pathologists).

### Ontario Schedule of Benefits (SOB) fees

The SOB fees increase closely tracked the W2Q numbers. This is not coincidental. The W2Q and SOB fees are so closely related; they cannot be considered independent measures. It should be noted that there are no actual billings to compare with the herein calculated SOB fees. Compensation in the laboratory is almost entirely based on the province’s “uniform level of compensation” for pathologists (which is paid in a complex arrangement by the hospital and Ministry of Health). The fee schedule is used for/in (1) referred in “RR cases”/cases sent to the lab for review/further analysis (not analyzed herein), and (2) non-hospital laboratories; these predominantly process smaller specimens (e.g. L864s).

### Measuring workload/capturing complexity

The traditional workload measures (case count, billings) are misleading, as there has been a shift in the case mix and the complexity of cases have increased. Report length captures the amount of information within the report and has been found to be representative of work over a twenty year period [24]. The block count is an indirect measurement of the work [1]. It is impossible to manipulate in small (biopsy) specimens; however, it can be in large resections. A high volume of ancillary testing does not necessarily represent added value [23]. It is noted that the Royal College of Pathologist (UK) workload system has a built-in disincentive for more blocks [4].

The number of report lines / (non-space) characters appears to be a robust measure but may be manipulated or “gamed”. Thus, the number of required reporting elements may be worth exploring as a workload measure. Currently, synoptic reports (Cancer Care Summaries) in W2Q and L4E are all weighted equally (1 W2Q unit, 3 L4E units).

If a mandated synoptic report has 25 separate report elements (or questions) and another has 5 questions, the former is likely more work than the latter. The weighting could be adjusted by the required ancillary testing (e.g., MSI testing in endometrial cancer)/ancillary testing recommended by consensus statements (elastin staining to assess for lymphatic invasion/vascular invasion in colorectal cancer resections).

Work complexity is important to consider in the larger context of workforce planning. The local workforce (as measured by number of FTEs) has not changed substantially in the past nine years; however, the Canadian pathologist workforce has increased significantly. In the time period 2007 to 2017, the Canadian pathology workforce increased by approximately 20% [25]. Though country to country workforce comparisons may be compounded by several factors and of limited value [16], in the same time period (2007 to 2017), the US pathology workforce decreased by approximately 18% [25].

### Technical changes and progress

Pathology may be in a unique position at this time vis-à-vis other specialties. If the pathology case complexity (conservatively estimated) has increased 25% and a pathology case can be considered analogous to a patient encounter, does it take other specialists 25% longer for a patient encounter versus 9 years ago?

The analysis herein presents several metrics that are broadly congruent and demonstrate increasing work for hospital pathologists. As a group, the authors believe the increased reporting requirements have improved care and represent progress; however, this has also been an erosion of the practice environment in hospital pathology. If the workforce had kept pace with the workload, approximately 6–7 full-time equivalent (anatomic) pathologists would have been added to the team in the 9-year period analyzed.

## Conclusions

The study demonstrates with several measures that the clinical (service) work has increased considerably in the examined laboratory without a commensurate increase in the anatomic pathology workforce, as measured by FTEs. Seen broadly, this trend (if universal) may have significant adverse quality of care implications and make the recruitment of future talent to pathology more challenging and, due to time constraints, impede collaborative research with other physicians and surgeons.

Famed Canadian physician Dr. William Osler stated many years ago “as is our pathology, so is our practice” [26]. We believe the quote is still relevant today, especially with the paradigm shifting to personalized medicine, where a more precise diagnosis, classification and biomarkers are dependent on the pathologist performing their duties with sufficient resources. The unique challenges to the pathology work environment should be a concern, and prompt further local investment in the anatomic pathology workforce.

## Supporting information

S1 File Both S1 and S2 are contained with a compressed file (filename: “S1_and_S2__anondata4pub__Workload_Evol.7z”). (DOCX)

S2 File (DOCX)

S1 Data (7Z)
