# Supplementary material for: Evolution of anatomic pathology workload from 2011 to 2019 assessed in a regional hospital laboratory via 574,093 pathology reports
Source: PLoS One. 2021 Jun 29;16(6):e0253876. doi: 10.1371/journal.pone.0253876 (PMC8241038; doi:10.1371/journal.pone.0253876)
Supplement: S1 File — (DOCX) [file pone.0253876.s001.docx]

**S1 File. Both S1 and S2 are contained with a compressed file (filename: “S1_and_S2__anondata4pub__Workload_Evol.7z”).** That file can be uncompressed with a freely available software ( <https://www.7-zip.org/> ). S1 (filename: “S1_anondata4pub_cases.csv”) includes the parameters: Acc_Year (the year the case was accessioned by pathology), L4E_2018 (Level 4 Equivalent (2018) units), W2Q (Work2Quality units), SOBF (Schedule of Benefits fees), Blocks (number of blocks), IHC (number of immunohistochemical stains), SS (number of special stains), dxlines (number of lines in “diagnosis” section), microlines (number of lines in “microscopic” section), CCSlines (number of lines in “Cancer Care Summary” section), L861 (number of L861 codes), L862 (number of L862 codes), L863 (number of L863 codes), L864 (number of L864 codes), L865 (number of L865 codes), L866 (number of L866 codes); the data is ordered by the case numbers. S2 (filename: “S2_anondata4pub_fte_SORTED.csv”) includes the parameters: Pathologist (anonymized), SO_date (sign-out date (in YEARMMDD format); the date the pathologist placed the final signature on the case); this data was used to calculate the number of full-time equivalents (FTEs). The data is ordered by the (anonymous) pathologist identifier.
