# Supplementary material for: Evolution of anatomic pathology workload from 2011 to 2019 assessed in a regional hospital laboratory via 574,093 pathology reports
Source: PLoS One. 2021 Jun 29;16(6):e0253876. doi: 10.1371/journal.pone.0253876 (PMC8241038; doi:10.1371/journal.pone.0253876)
Supplement: S2 File — (DOCX) [file pone.0253876.s002.docx]

## Overview of Workload Systems

## Overview of Ontario Schedule of Benefits and Work2Quality Workload System

In Ontario, Canada, the government-run health plan, the Ontario Health Insurance Plan (OHIP), pays for medical services deemed necessary. The health insurance act provides the legal foundation and a framework for the schedule of benefits for physician services. The schedule lists services insured by OHIP and includes a general preamble that applies to all physicians and subsections that apply various specialties.

The unit of a service in surgical pathology is a specimen. A specimen is tissue that is identified for individual and separate examination and diagnosis. The fee schedule is based on the complexity of the specimen and the description and fees for the most common specimens and tests is summarized below.

**S1 Table: Selected L Codes (Commonly Used in Anatomical Pathology) with Fee Amount (in Canadian Dollars) and Work2Quality (W2Q) Weighting**

| L Code | Description | Fee | W2Q |
| --- | --- | --- | --- |
| L861 | Gross examination only without microscopic examination | $5.20 | 0.11 |
| L862 | Appendix (incidental appendectomy); fallopian tube (sterilization); digit (traumatic amputation); hernia sac; hydrocele sac; nerve; skin (neonatal foreskin; plastic repair); sympathetic ganglion; testis (castration); vaginal mucosa (incidental); vas deferens (sterilization) | $8.45 | 0.17 |
| L863 | Abscess; aneurysm; anal tag; appendix (other than incidental); artery or vein (ather*oma*tous plaque; varicosity); Bartholin gland cyst; bone (other than pathologic fracture); bursa or synovial cyst; carpal tunnel tissue; cartilage (shavings); cholesteat*oma*; colostomy st*oma*; conjunctiva (pterygium); cornea; diverticulum (digestive tract); Dupuytren contracture tissue; femoral head (other than fracture); fissure or fistula; gallbladder; ganglion cyst; haemat*oma*; haemorrhoid; hydatid of Morgagni; intervertebral disc; joint loose body; meniscus; mucocele (salivary); neur*oma* (traumatic; Morton); nasal or sinusoidal polyp (inflammatory); skin (acrochordon/tag; cyst; foreskin, other than neonate; debridement; pilonidal cyst or sinus); soft tissue (lip*oma*, debridement); spermatocele; tendon or tendon sheath; testicular appendage; thrombus or embolus; uterine contents (induced abortion); varicocele; vas deferens (other than sterilization). | $14.30 | 0.29 |
| L864 | Artery (b*iop*sy); bone marrow (b*iop*sy); bone exostosis; brain or meninges (other than neoplasm resection); branchial cleft cyst; breast (b*iop*sy, not requiring microscopic evaluation of surgical margin; reduction mammoplasty); bronchus (b*iop*sy); cell block; cervix (b*iop*sy); digestive tract (b*iop*sy); endocervix (b*iop*sy or curettings); endometrium (b*iop*sy or curettings); extremity (traumatic amputation); fallopian tube (b*iop*sy; ectopic pregnancy); femoral head (fracture); digit (non-traumatic amputation); heart valve; joint (resection); kidney (b*iop*sy); larynx (b*iop*sy); lip (b*iop*sy; wedge resection); lung (transbronchial b*iop*sy); lymph node (b*iop*sy); muscle (b*iop*sy); nasal mucosa, nasopharynx or oropharynx (b*iop*sy); nerve (b*iop*sy); odontogenic or dental cyst; omentum (b*iop*sy); oral or gingival mucosa (b*iop*sy); ovary *with or without* fallopian tube (non-neoplastic); ovary (b*iop*sy, wedge resection); paranasal sinus (b*iop*sy); parathyroid gland; pericardium (b*iop*sy); peritoneum (b*iop*sy); pituitary gland (neoplasm); placenta (other than third trimester); pleura (b*iop*sy); polyp (cervical; endometrial; digestive tract); prostate (needle b*iop*sy; transurethral resection); salivary gland (b*iop*sy); skin (other than cyst / tag / debridement / plastic repair); synovium; spleen; testis (other than b*iop*sy, castration or neoplasm); thyroglossal duct cyst; tongue (b*iop*sy); tonsil or adenoid (b*iop*sy); trachea (b*iop*sy); ureter (b*iop*sy); urethra (b*iop*sy); urinary bladder (b*iop*sy); uterine contents (spontaneous or missed abortion); uterine leiomy*oma* (myomectomy); uterus *with or without* tubes and ovaries (for prolapse); vagina (b*iop*sy); vulva (b*iop*sy). | 48.65 | 1.00 |
| L865 | Adrenal gland (resection); bone (b*iop*sy or curettings, pathologic fracture); brain (b*iop*sy); brain or meninges (neoplasm resection); breast (partial or simple mastectomy; excision requiring microscopic evaluation of surgical margin); cervix (conization); colon (segmental resection, other than neoplasm); extremity (non-traumatic amputation); eye (enucleation); kidney (partial or total nephrectomy); larynx (partial or total resection); liver (b*iop*sy or wedge or partial resection); lung (wedge b*iop*sy); lymph nodes (regional resection; sentinel); mediastinum (b*iop*sy); myocardium (b*iop*sy); odontogenic neoplasm; ovary *with or without* fallopian tube (neoplasm); pancreas (b*iop*sy); placenta (third trimester); prostate (other than transurethral resection or radical resection); salivary gland; small intestine (resection, other than neoplasm); soft tissue mass (other than lip*oma*; b*iop*sy or simple excision); st*oma*ch (partial or total resection, other than neoplasm); testis (b*iop*sy); thymus (neoplasm); thyroid (partial or total thyroidectomy); ureter (resection); urinary bladder (transurethral resection); uterus *with or without* fallopian tubes and ovaries. | $103.20 | 2.12 |
| L866 | Bone (resection); breast (mastectomy with regional lymph nodes); colon (segmental resection for neoplasm); colon (total resection); extremity (disarticulation); fetus (with dissection); larynx (partial or total resection with regional lymph nodes); lung (partial or total resection); oesophagus (partial or total resection); pancreas (partial or total resection); prostate (radical resection); small intestine (resection for neoplasm); soft tissue neoplasm (extensive resection); st*oma*ch (partial or total resection for neoplasm); testis (neoplasm); tongue (resection for neoplasm); tonsil (resection for neoplasm); urinary bladder (partial or total resection); uterus *with or without* fallopian tubes and ovaries (neoplasm other than leiomy*oma*); vulva (partial or total resection) | $181.65 | 3.73 |
| L822 | Operative consultation, with or without frozen section | $77.20 | 1.59 |
| L823 | Each subsequent frozen section or direct smear | $38.25 | 0.79 |
| L837 | Immunohistochemistry and interpretation-per marker | $15.60 | 0.32 |
| L868 | Special histochemistry for identification of microorganisms | $35.05 | 0.72 |
| L869 | Special histochemistry for identification of elements other than microorganisms | $15.55 | 0.32 |

## Overview of Level 4 Equivalent (2018)

The Level 4 Equivalent (L4E) Workload System is based on worked published by Dr. Raymond Maung[1] in 2005 and was adopted by the Canadian Association of Pathologists.

Since its adoption, the model has been regularly updated regularly every 4-5 years to keep the model current and correct deficiencies by a Committee of the Canadian Association of Pathologists – the “Workload and Human Resources Committee” ( <https://cap-acp.org/wkload.php> ). It is now inclusive of all pathologist associated activities in Anatomic and hematopathology (consultation, quality assurance activities including various types of consults, administration and medical oversight, and academic – teaching, mentoring, training and research) and flexible to accommodate the presence/absence of pathologist assistants, cytotechnologists and trainees. The most recent update was done in 2018 and model for hematopathology and transfusion medicine included in 2019. It is available at the Canadian Association of Pathologist website (see below). There is a companion Microsoft Excel workbook to document the workload of a department and calculate the appropriate Full Time Equivalents (FTE) needed; it is available here: [https://cap-acp.org/cmsUploads/CAP/File/CAP-ACP%20human%20resources%20WorkBook%20%2020200721.xlsx](https://cap-acp.org/cmsUploads/CAP/File/CAP-ACP%20human%20resources%20WorkBook%2020200721.xlsx)

One L4E unit represents approximately 10 minutes of work. L4E points are generally assigned based on time associated with signing out the case, the medical value to clinicians and patients, the clinical urgency, the degree of difficulty and the medico-legal responsibility. In the latest version, most specimens can be coded using nine rules that cover grossing, routine microscopic assessment, ancillary testing and synoptic reporting. Consultations and various QA activities, multidisciplinary conferences, administrative/medical oversight duties, teaching and research are also assigned point values. The official L4E definition is here: [https://cap-acp.org/cmsUploads/CAP/File/CAP-ACP%20Workload%20AP%20%20HP%20Model%20%2020200801.pdf](https://cap-acp.org/cmsUploads/CAP/File/CAP-ACP%20Workload%20AP%20HP%20Model%2020200801.pdf)

An overview on a Canadian-based pathology wiki is here: <https://librepathology.org/wiki/Level_4_equivalent_of_2018>
